# Machine learning models for predicting postoperative acute kidney injury in pediatric cardiac surgery: a systematic review and meta-analysis

**DOI:** 10.3389/fcvm.2026.1808152

**Published:** 2026-06-18

**Authors:** Noel Matthew Imaniku Sihombing, Hashfi Fauzan Raz, Sandra Rosa Uli Siahaan, Shinaya Yemia Retra Sihombing, Ahmad Dwi Rifa’i, Muhammad Hikmal Duha

**Affiliations:** 1 Faculty of Medicine, Universitas Sumatera Utara, Medan, Indonesia; 2 Division of Thoracic, Cardiac, and Vascular Surgery, Department of Surgery, Faculty of Medicine, Universitas Sumatera Utara, Haji Adam Malik General Hospital, Medan, Indonesia

**Keywords:** acute kidney injury, artificial intelligence, cardiac surgery, machine learning, pediatric

## Abstract

**Background:**

Acute kidney injury (AKI) occurs in up to 42% of pediatric cardiac surgeries and is associated with prolonged intensive care, increased morbidity and in-hospital mortality. Machine learning (ML) has emerged as a promising approach for early AKI risk stratification by modeling complex, high-dimensional clinical data.

**Objectives:**

To systematically review and meta-analyze the diagnostic accuracy of ML models for predicting pediatric cardiac surgery associated AKI (CSA-AKI).

**Methods:**

We performed a systematic search of PubMed, ScienceDirect, Springer, and DOAJ. The review protocol was prospectively registered in PROSPERO (CRD420251145645). Study quality was assessed using QUADAS-2 tool and PROBAST + AI. A bivariate random-effects diagnostic meta-analysis was performed using Stata 17.0 to estimate the pooled area under the summary receiver operating characteristic curve (SROC AUC), sensitivity, specificity, likelihood ratios, and diagnostic odds ratio (DOR).

**Results:**

A meta-analysis of seven studies yielded a pooled SROC AUC of 0.91 (95% CI 0.88–0.93), driven predominantly by internally validated models (AUC 0.93, Sensitivity of 0.84, Specificity of 0.95). Externally validated models showed substantially lower performance (Sensitivity 0.70, Specificity 0.80), representing the more clinically relevant benchmark. A sensitivity analysis using median-performing models confirmed directional consistency (AUC 0.85, Sensitivity 0.75, Specificity 0.91). Substantial heterogeneity was observed (*I*^2^ = 81.48%).

**Conclusion:**

ML models show promising accuracy for predicting pediatric CSA-AKI. Substantial heterogeneity and limited external validation warrant cautious interpretation and further multicenter validation before clinical use.

**Systematic Review Registration:**

https://www.crd.york.ac.uk/PROSPERO/view/CRD420251145645.

## 1 Introduction

Acute kidney injury (AKI) is a prevalent and clinically significant cardiac surgery complication among pediatric patients, with incidence rates reaching up to 42% of pediatric patients undergoing cardiac surgery with mortality rates ranging from 20% to 79% (1). The pathogenesis of AKI following cardiac surgery is multifactorial, with renal ischemia, reperfusion injury, systemic inflammation, and hemolysis from cardiopulmonary bypass (CPB) playing central roles (2, 3). Furthermore, pediatric patients who underwent cardiac surgery often present with prolonged cyanosis, which serve as predisposing factors for the development of AKI (4). Children who develop AKI after cardiac surgery experience prolonged intensive care unit and hospital stays, reduced quality of life, and increased in-hospital mortality. In severe cases, they often require renal replacement therapy (5).

Early diagnosis and appropriate management during the perioperative period are key to reducing mortality, morbidity, and long-term renal damage associated with acute kidney injury (6). Currently, there are no specific guidelines regarding cardiopulmonary bypass strategies to prevent AKI in the pediatric population (7). In recent years, several predictive models combining biomarkers and clinical variables have been developed to predict the occurrence of cardiac surgery-associated acute kidney injury (CSA-AKI) in children. However, these models are limited by small sample sizes, lack of both internal and external validation, and increased financial burden due to the use of novel biomarkers (8, 9).

With the advancement of technology, artificial intelligence, especially machine learning (ML), offers powerful tools to enhance predictive capabilities by analyzing and interpreting complex datasets, identifying recurring patterns, and building data-driven predictive models. A meta-analysis has demonstrated the capability of ML approaches in accurately predicting CSA-AKI in adults, showing promising performance in early detection (10), but their predictive performance for CSA-AKI in the pediatric population has been rarely evaluated. The main diseases, causes, and risk factors of CSA-AKI in children are quite different from those in adults, so the prediction models used for adults cannot be directly applied to pediatric patients (11).

To address this gap, we conducted a systematic review and meta-analysis to assess the predictive performance of machine learning algorithms for acute kidney injury following cardiac surgery aiming to provide evidence-based support for their clinical implementation.

## 2 Methods

The protocol used in compiling this meta-analysis follows the guidelines of the Preferred Reporting Items for Systematic Review and Meta-analysis (PRISMA). This systematic review and meta-analysis was prospectively registered in the International Prospective Register of Systematic Reviews (PROSPERO; registration number CRD420251145645).

### 2.1 Search strategy and study selection

A systematic search of the literature was conducted using electronic databases, namely PubMed, ScienceDirect, Springer, and DOAJ. The search strategy can be seen in the Supplementary File. The studies included in this systematic review are those involving pediatric patients who underwent cardiac surgery, according to the prespecified inclusion and exclusion criteria, with no restriction on the year of publication. The inclusion criteria for samples include: (1) Randomized controlled trial (RCT), prospective cohort study, nested case-control study, case-control study on pediatric patients who had undergone cardiac surgery, (2) English language, (3) Publication in a journal with a full text online, (4) Studies that established a complete predictive model using machine learning. We excluded studies that predicted AKI without applying machine learning methods, included mixed adult and pediatric populations, or did not report relevant outcome measures.

### 2.2 Data extraction and quality assessment

To avoid the risk of assessment bias, this systematic review involves two reviewers who independently assess the obtained studies. The first reviewer is NMIS, and the second reviewer is SRUS. Differences in review results between the first and second reviewers are discussed together until a consensus is reached.

The quality of included studies was assessed using the Quality Assessment of Diagnostic Accuracy Studies (QUADAS-2) tool, which evaluates the risk of bias and applicability across four key domains: patient selection, index test, reference standard, and flow and timing. In addition to QUADAS-2, risk of bias and applicability of the prediction models were assessed using the Prediction model Risk of Bias Assessment Tool (PROBAST). PROBAST evaluates four domains (participants, predictors, outcome, and analysis) and provides an overall judgment of low, high, or unclear risk of bias.

Performance metrics were extracted from the test set as reported in the primary studies. When a study reported both internal and external validation the external validation result was preferentially extracted. To avoid unit of analysis error when multiple machine learning models were developed from the same patient cohort only the single best performing model was included in the primary meta-analysis. This model was defined in advance as the one with the highest AUC in the external validation or independent test set. If a study did not clearly designate a primary model the best performing validated model was selected. All other models from the same cohort were excluded from the main analysis.

### 2.3 Statistical analysis

Meta-analysis was performed using Stata 17.0 (StataCorp, College Station, TX, USA). True positive (TP), false positive (FP), false negative (FN), and true negative (TN) were extracted from each study to construct four-fold tables. A bivariate random-effects model was used to jointly pool sensitivity, specificity, positive likelihood ratio (PLR), negative likelihood ratio (NLR), diagnostic odds ratio (DOR) with 95% confidence intervals while accounting for their negative correlation. Because most studies did not report the exact probability threshold used, the Youden index was assumed when not explicitly stated. The summary receiver operating characteristic area under the curve (SROC AUC) was pre-specified as the primary outcome measure. When essential data were incomplete or not clearly reported, the researchers contacted the corresponding authors of the selected studies to obtain clarification or missing information. The specificity and sensitivity of ML combinations from different studies were assessed using forest plots. Statistical heterogeneity was evaluated by Cochrane's *Q* test and *I*^2^ statistic, with subgroup analyses conducted to explore sources of heterogeneity. Deeks' funnel plot asymmetry test (*p* > 0.05) was used to assess publication bias. Subgroup analyses were limited to algorithm families reported in at least two independent studies and were considered exploratory because of the small number of studies per subgroup. Random-effects models were used throughout. For the LGBM subgroup, a bivariate random-effects model was applied. For subgroups with fewer than four studies, the bivariate model did not converge, so univariate REML models were used separately for sensitivity and specificity.

## 3 Results

### 3.1 Study characteristics

**Figure 1:**
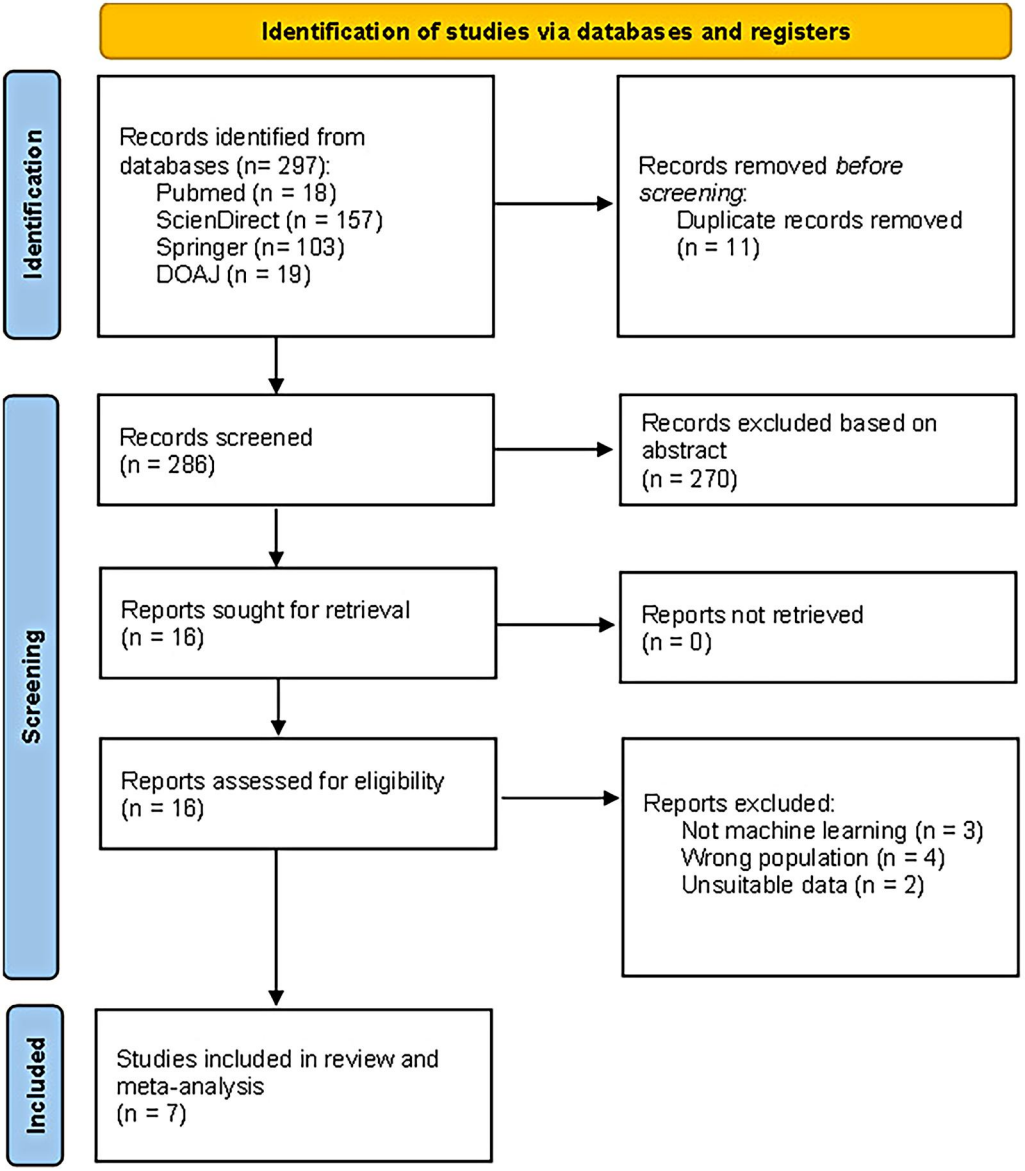
Flowchart of the included patients in the analysis.

A total of 297 articles were identified from PubMed, ScienceDirect, Springer, and DOAJ. After removing 11 duplicates, the titles and abstracts of the remaining 286 articles were screened. Sixteen full-text studies were assessed for eligibility, and finally, seven studies were included in the meta-analysis. The flowchart of study selection and identification is presented in Figure 1.

A total of seven studies were included in the meta-analysis. All included studies were retrospective cohort designs conducted between 2022 and 2026. The meta-analysis included a total of 11,867 pediatric participants undergoing cardiac surgery, of whom 686 developed AKI and 11,181 did not. Most studies were conducted in China (57%), with two originating from USA and one from Italy. The median age across all studies was below 5 years, and participants were predominantly male in half of the studies (with male percentages ranging from 44.02% to 67.91%). The baseline characteristics of the included studies are summarized in Table 1.

**Table 1:** Characteristics of the included studies.

Author	Country	Study	Case/Normal	Age (month)	Male (%)	ML algorithm	AKI lead time prediction
Fragasso et al. (12)	Italy	Single center retrospective	223/196	5.47 (1–33.3)	53.93%	RF^a^^,^b^^	48 h post-surgery
Tong et al. (13)	China	Single center retrospective	147/8,926	11.6 (5.2–32.7)	52.50%	LR	Follow-up until discharge
						RF^b^	
						LGBM^a^	
						SVM	
						CatBoost	
Kong et al. (7)	China	Single center retrospective	67/67	AKI: 2.0 (1.0–7.0)	67.91%	LR^a^	7 postoperative days
						LGBM	
						SVM^b^	
				Non-AKI: 3.0 (1.5–9.6)		GNB	
						XGB	
						MLP	
Luo et al. (11)	China	Multicenter retrospective	51/534	48.0 (12.0–96.0)	49.23%	XGB^a^^,^b^^	7 postoperative days
Zeng et al. (14)	China	Single center retrospective	133/1,222	11.9 (4.5–28.9)	49.37%	LR	24 h post-surgery
						SVM	
						LSTM	
						GRU^b^	
						Dipole^b^	
						RETAIN	
						TAARNN^a^	
Nagy et al. (15)	USA	Single center retrospective	14/67	6.0 (2.0–27.0)	44.02%	LGBM^a^^,^b^^	48 h post-surgery
Baloglu et al. (16)	USA	Multicenter retrospective	51/169	10.3 (2.8–52.0)	56.5%	LGBM^a^	7 postoperative days
						XGB	
						CatBoost^b^	
						HGB	

LR, logistic regression; RF, random forest; LGBM, light gradient boosting machine; SVM, support vector machine; CatBoost, categorical boosting; GNB, Gaussian naive Bayes; XGB, extreme gradient boosting; HGB, histogram-based gradient boosting; MLP, multilayer perceptron; LSTM, long short-term memory; GRU, gated recurrent unit; RETAIN, reverse time attention; TAARNN, time-aware attention-based recurrent neural network.

a Best-performing model selected for primary meta-analysis based on highest AUC.

b Median-performing model selected for sensitivity analysis based on median AUC.

Regarding AKI grade prediction, the studies varied in their focus: three targeted severe AKI (defined as KDIGO stages 2–3), three addressed all stages of AKI (per KDIGO criteria), and one specifically predicted renal failure (also based on KDIGO). For AKI lead time prediction, the timelines differed across studies, including 24 h post-surgery, 48 h post-surgery 7 days post-surgery, postoperative ICU stay, and 2 days post-surgery, and follow-up until discharge, reflecting diverse approaches to early detection and monitoring in pediatric cardiac surgery patients.

### 3.2 Risk of bias

The risk-of-bias assessment using the QUADAS-2 tool.is summarized in Supplementary Figure S1. Overall, most domains demonstrated a low risk of bias. Detailed QUADAS-2 assessment is provided in Supplementary File.

PROBAST + AI assessment (Table 2) showed that five studies had low overall risk of bias, while the remaining two studies (Fragasso 2023 and Kong 2023) had high risk of bias, primarily due to lack of external validation and potential data leakage in the analysis domain.

**Table 2:** Risk of bias assessed by using the PROBAST + AI tool.

Author	Risk of bias				Applicability			Overall	
	Participants	Predictor	Outcome	Analysis	Participants	Predictors	Outcome	Risk of Bias	Applicability
Fragasso et al. (12)	+	+	+	−	+	+	+	−	+
Kong et al. (7)	+	+	+	−	+	+	+	−	+
Luo et al. (11)	+	+	+	+	+	+	+	+	+
Nagy et al. (15)	+	+	+	+	+	+	+	+	+
Tong et al. (13)	+	+	+	+	+	+	+	+	+
Zeng et al. (14)	+	+	+	+	+	+	+	+	+
Baloglu et al. (16)	+	+	+	+	+	+	+	+	+

(−) high risk.

(+) low risk.

### 3.3 Diagnostic performance of machine learning

**Figure 2:**
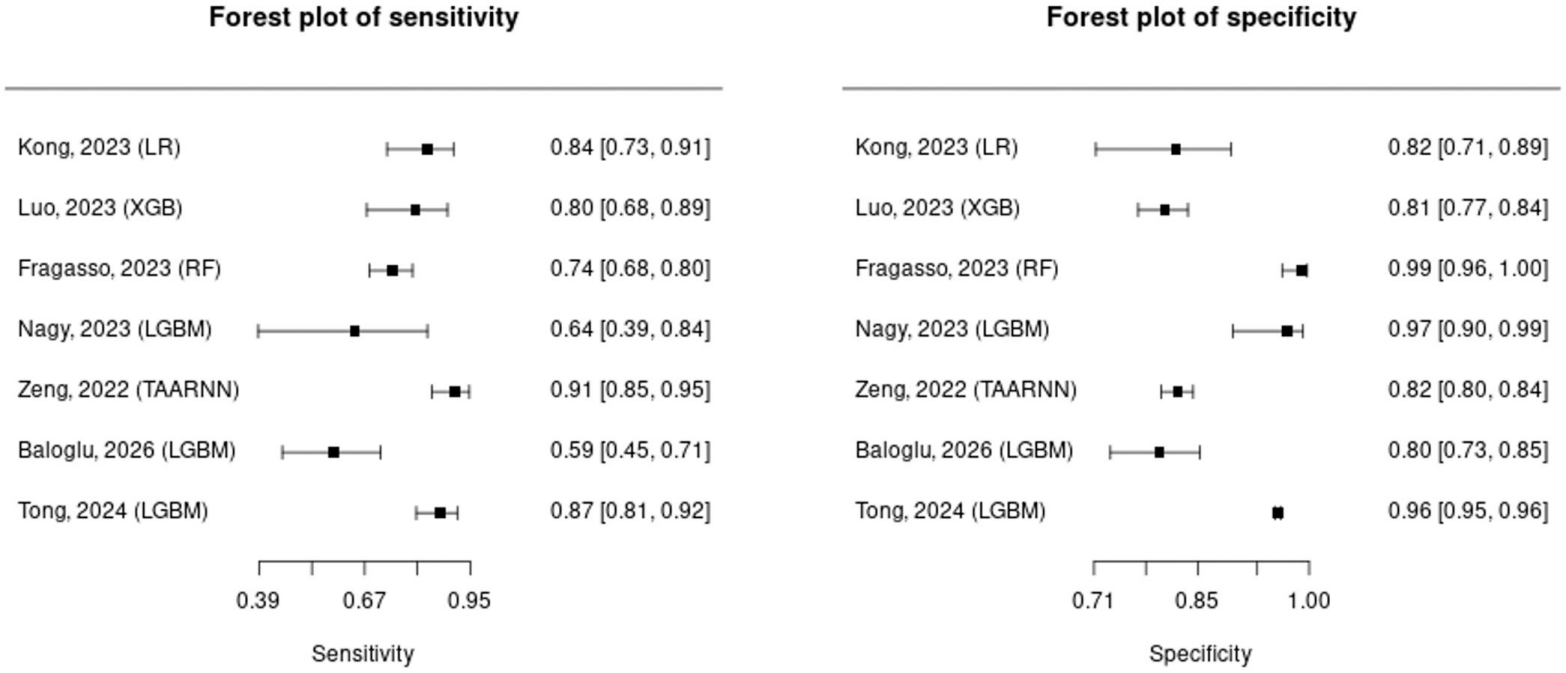
Forest plot of pooled sensitivity and specificity.

**Figure 3:**
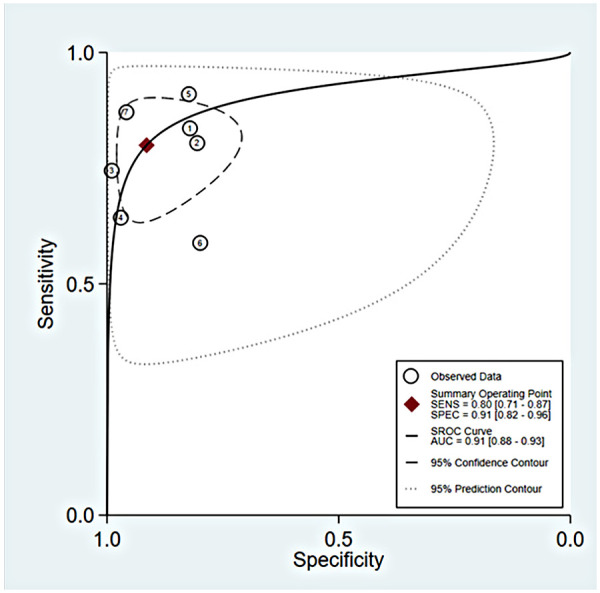
SROC AUC.

A meta-analysis of seven studies using a bivariate random-effects model yielded a pooled SROC AUC of 0.91 (95% CI 0.88–0.93), with pooled sensitivity of 0.80 (95% CI 0.71–0.87) and specificity of 0.91 (95% CI 0.82–0.96) (Figures 2, 3). The corresponding summary point on the hierarchical summary receiver operating characteristic (HSROC) curve showed a DOR of 42.6 (95% CI 16.95–107.10), a PLR of 9.35 (95% CI 4.32–20.24), and a NLR of 0.21 (95% CI 0.15–0.32). The HSROC parameters demonstrated a lambda value of 3.62, reflecting high diagnostic accuracy, while the beta coefficient was not statistically significant (*β* = 0.64, *p* = 0.166), suggesting minimal asymmetry and no strong evidence of a threshold effect. Substantial heterogeneity was observed (*I*^2^ = 81.48% for AUC). Sensitivity analysis excluding the extremely imbalanced Tong et al. study (147 AKI vs. 8,926 non-AKI) yielded nearly identical results (sensitivity 0.78, specificity 0.90), indicating robustness to class imbalance (see Supplementary File).

To address potential selection bias, a sensitivity analysis was performed using the median-performing model based on AUC. The results remained robust: sensitivity 0.745 (95% CI 0.670–0.809), specificity 0.907 (95% CI 0.800–0.960), DOR 28.52 (95% CI 10.05–80.96), PLR 8.01 (95% CI 3.47–18.48), and NLR 0.28 (95% CI 0.21–0.38). The beta coefficient for the threshold effect was 1.04 (*p* = 0.033) (see Supplementary File).

Stratified analysis by validation type showed a substantial difference. Among the five studies employing internal validation, the pooled SROC AUC was 0.93 (95% CI 0.90–0.95), with pooled sensitivity of 0.84 (95% CI 0.77–0.89) and specificity of 0.95 (95% CI 0.86–0.98). The DOR was 97.71 (95% CI 42.04–227.10), PLR was 16.46 (95% CI 6.03–44.88), and the NLR was 0.17 (95% CI 0.12–0.24). Heterogeneity within this subgroup was markedly lower than the overall pool (*I*^2^ = 26.67%). Only two studies performed true external validation on independent cohorts from distinct institutions or time periods. Across these two externally validated studies, the observed pooled sensitivity was 0.70 (95% CI 0.46–0.87) and specificity was 0.80 (95% CI 0.77–0.83) (see Supplementary File).

### 3.4 Meta-regression and subgroup analyses

**Figure 4:**
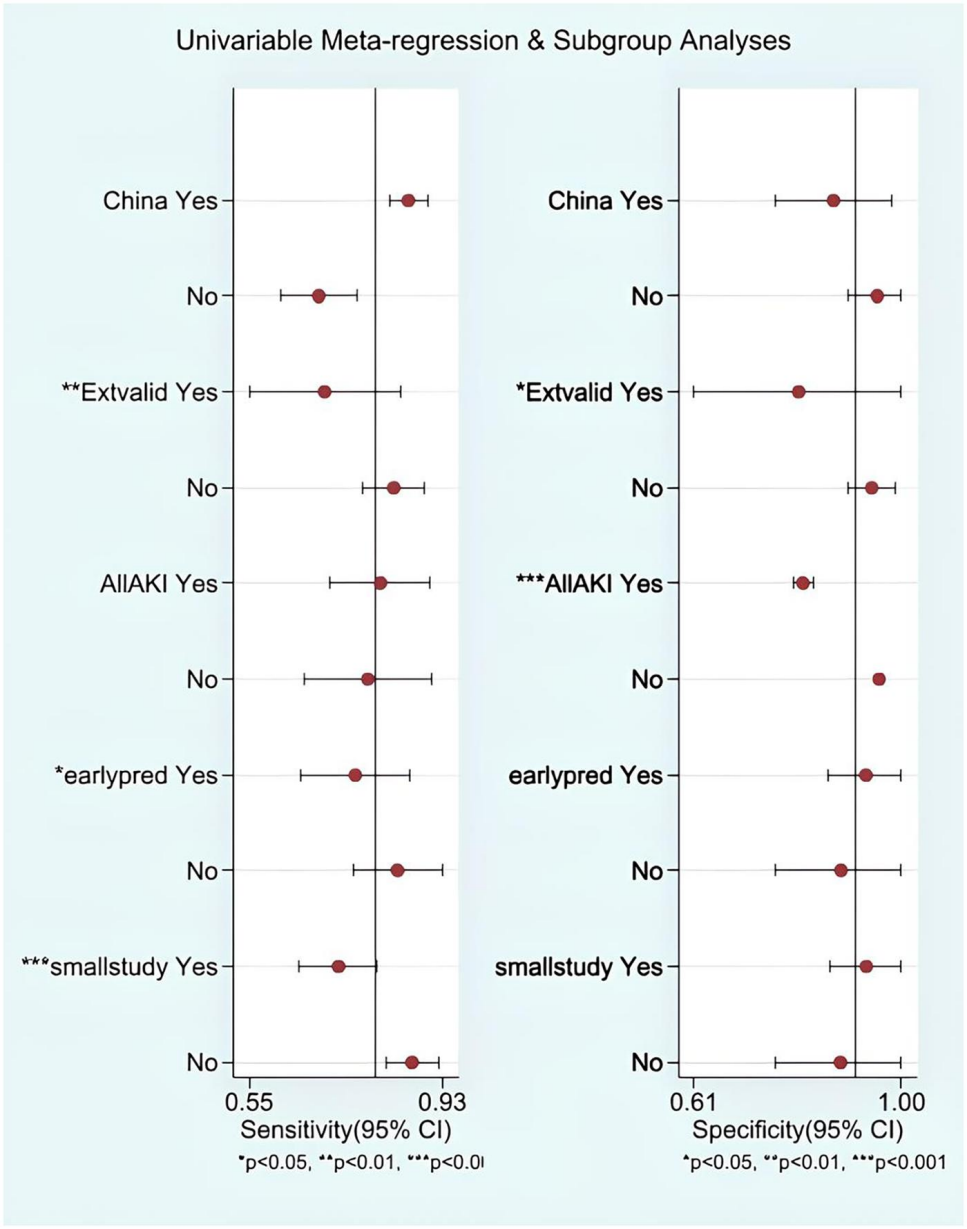
Meta-reggression and subgroup analysis.

**Figure 5:**
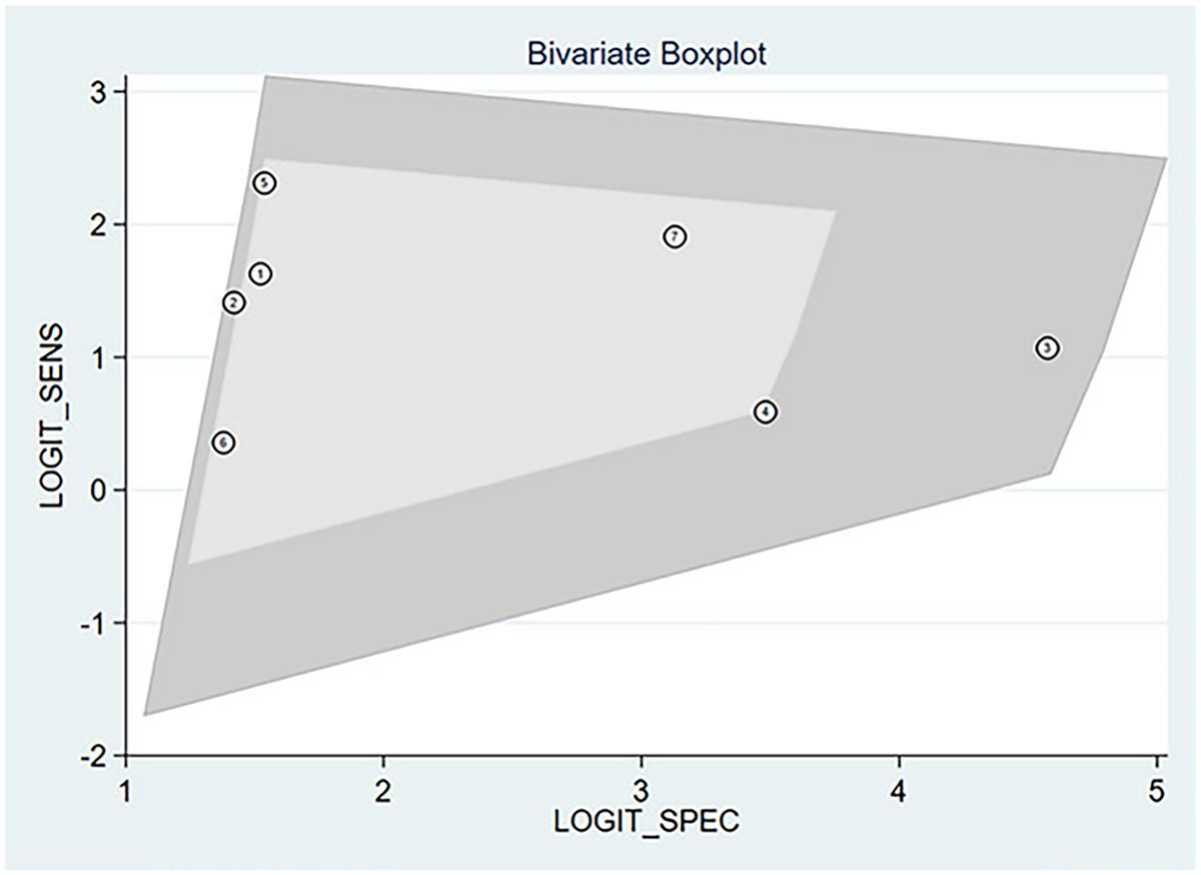
Bivariate boxplot.

Meta-regression was performed to explore sources of heterogeneity using study-level covariates: country of origin (China vs. non-China), validation type (external vs. internal), AKI definition (any-stage vs. severe AKI), prediction time window (early ≤48 h vs. later), study size (small vs. large) (Figure 4). Study region was a significant modifier (LRT *χ*^2^ = 13.51, *p* < 0.001), with studies conducted in China demonstrating higher sensitivity (0.87, 95% CI 0.83–0.91) but lower specificity (0.87, 95% CI 0.76–0.98) than studies from other regions (*n* = 3). External validation significantly reduced performance (LRT *χ*^2^ = 8.04, *p* = 0.02), as externally validated models (*n* = 2) showed lower sensitivity (0.70, 95% CI 0.55–0.85) and specificity (0.81, 95% CI 0.61–1.00) compared with internally validated models. AKI definition was also a strong modifier (LRT *χ*^2^ = 23.73, *p* < 0.001): studies predicting any-stage AKI (*n* = 4) had higher sensitivity but lower specificity (0.81, 95% CI 0.79–0.83) than those predicting severe AKI. Smaller studies (*n* = 4) were associated with lower sensitivity (0.73, 95% CI 0.65–0.80; LRT *χ*^2^ = 6.12, *p* = 0.05). In contrast, prediction time window (*p* = 0.44) were not significant sources of heterogeneity. A bivariate boxplot was used to visually inspect outlying studies to assess potential contributors to heterogeneity (Figure 5).

LightGBM was evaluated in four independent studies and analyzed using a bivariate random-effects model, yielding a pooled sensitivity of 0.72 (95% CI 0.56–0.84), specificity of 0.90 (95% CI 0.81–0.95) and DOR of 23.91 (95% CI 5.71–100.07). Logistic regression, XGBoost, and SVM were each evaluated in three studies, while Random Forest and CatBoost were each assessed in two studies (see Table 3). These subgroup results are exploratory and should be interpreted with caution given the limited number of studies per subgroup.

**Table 3:** Algorithm subgroup summary based on studies available for each algorithm family.

Algorithm	Studies	Sensitivity (95% CI)	Specificity (95% CI)
LGBM^a^	4	0.72 (0.56–0.84)	0.90 (0.81–0.95)
LR	3	0.81 (0.71–0.88)	0.87 (0.66–0.96)
SVM	3	0.82 (0.62–0.93)	0.78 (0.73–0.83)
XGB	3	0.74 (0.60–0.85)	0.79 (0.76–0.82)
RF	2	0.81 (0.65–0.91)	0.97 (0.91–0.99)
CatBoost	2	0.75 (0.37–0.93)	0.91 (0.70–0.98)

a Subgroup analysis was performed using a bivariate random-effects model only for LightGBM-based algorithms. Other subgroups were analyzed using a univariate random effects model (REML).

### 3.5 Predictor

Table 4 presents the top ten predictors from four studies, showing notable variability. Common preoperative factors include baseline creatinine, age, eGFR, weight, and gender; intraoperative factors include CPB duration, ventilation time, and operation time; postoperative factors include creatinine and lactate. Most studies used SHAP (Shapley Additive Explanations) to identify key risk contributors, enhancing clinical interpretability.

**Table 4:** Predictor of postoperative AKI in pediatric cardiac surgery.

Study	Variables	Preoperative	Intraoperative	Postoperative
Fragasso et al. (12)	37	Basal creatinine	CPB duration (min)	Creatinine
				Platelets
				LDH
				Diuresis
				Ethacrynic acid
				aPTT
				BE
				Lactate
Tong et al. (13)	39	STAT score	Mechanical ventilation time (min)	–
		Oxygen support	Operation time (min)	
		RACHS-1	Anesthesia time (min)	
		ABC score	Mechanical ventilation time classification	
		Hematocrit		
		Age		
Kong et al. (7)	16	Cyanosis	Duration of renal ischemia (min)	–
		eGFR	Operation time (min)	
		Opening diameter of PDA	CPB strategy (MHCA/DHCA)	
		Weight (kg)		
		Newborn		
		Premature		
		Gender		
Luo et al. (11)	27	Basal creatinine	Perfusion time (min)	–
		Body length (cm)	Operation time (min)	
		eGFR	Intraoperative blood loss (mL/kg)	
		Potassium		
		Age (year)		
		Weight (kg)		
		Calcium		
Zeng et al. (14)	83	Length of stay (days)	Autologous blood transfusion (mL)	–
			Mechanical ventilation time (h)	
			FFP transfusion (mL)	
			Pressure of oxygen	
			Aortic cross clamp time (min)	
			C-reactive protein	
			Platelet count	
			CPB duration (min)	
			Pulse	
Nagy et al. (15)	34	Basal creatinine	Operation time (min)	POD0 serum pH
		Gender	CPB duration (min)	POD0 lactate
		Weight (kg)		POD0 vasoactive inotropic score
				POD0 hematocrit
				POD0 Creatinine
Baloglu et al. (16)	17	Basal creatinine	CPB duration (min)	–
		Weight (kg)	Aortic cross clamp time (min)	
		STAT score	Open sternum surgery	
		Age	Heart transplant surgery	
		Gender		
		Preterm		

CPB, cardiopulmonary bypass; POD0, postoperative day 0; LDH, lactate dehydrogenase; aPTT, activated partial thromboplastin time; BE, base excess; STAT, Society of Thoracic Surgeons–European Association for Cardio-Thoracic Surgery mortality score; RACHS-1, risk adjustment for congenital heart surgery-1; ABC, Aristotle basic complexity score; eGFR, estimated glomerular filtration rate; PDA, patent ductus arteriosus; MHCA, moderate hypothermic circulatory arrest; DHCA, deep hypothermic circulatory arrest; FFP, fresh frozen plasma.

### 3.6 Publication bias

**Figure 6:**
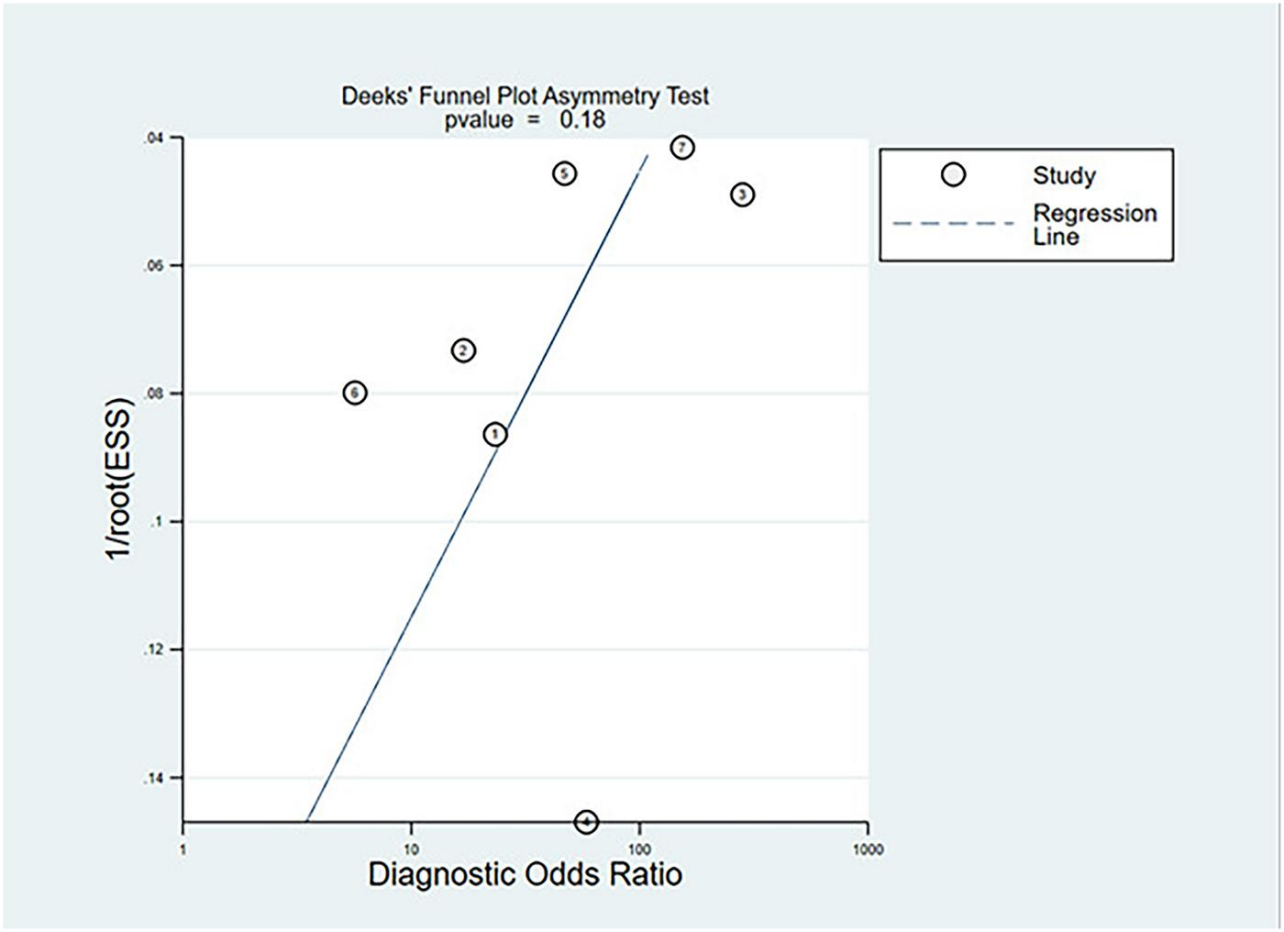
Deeks' funnel plot.

Deeks' funnel plot showed no clear asymmetry (*p* = 0.18); however, with only seven studies, the test is underpowered and the absence of publication bias cannot be conclusively ruled out (Figure 6).

## 4 Discussion

Early and accurate prediction of AKI after pediatric cardiac surgery is vital for improving outcomes, as traditional diagnostic approaches have notable limitations, emphasizing the need for novel predictive approaches (8). Machine learning, a subset of artificial intelligence, excels in analyzing electronic health record (EHR) data to detect nonlinear relationships and interactions among predictors, enabling robust CSA-AKI prediction in pediatric patients, as evidenced by several studies reporting high accuracy (14, 15, 17).

In this meta-analysis of seven studies involving 11,867 children, ML models demonstrated promising diagnostic performance, with a pooled SROC AUC of 0.91 (95% CI 0.88–0.93) as the primary outcome, sensitivity 0.80 (95% CI 0.71–0.87), specificity 0.91 (95% CI 0.82–0.96), and DOR 42.6 (95% CI 16.95–107.10). A sensitivity analysis using median-performing models confirmed directional consistency (Se 0.745, Sp 0.907, DOR 28.52), though a significant threshold effect emerged (*β* = 1.04, *p* = 0.033), suggesting best-model selection may partially obscure threshold-dependent behavior across studies. Critically, stratified analysis by validation type revealed a substantial performance gap: internally validated models yielded AUC 0.93, while externally validated models showed markedly lower sensitivity (0.70) and specificity (0.80). This transportability gap reflects overfitting to local data and institutional characteristics, and consistent with Scott et al., external validation estimates represent the more clinically relevant benchmark for real-world implementation (18).

ML-based prediction compares favorably against established non-ML approaches for pediatric CSA-AKI. Biomarker-based approaches have been extensively evaluated, with urinary NGAL-to-creatinine ratio demonstrating sensitivity of 91.3% and specificity of 89.7% (9), but ML models surpass urinary L-FABP (AUC 0.77) (19). ML offers advantages like real-time, non-invasive integration of diverse data, cost-effectiveness, and practicality for clinical use. Furthermore, ML exhibited superior performance in predicting AKI compared to nomograms (AUROC 0.755) and other AI approaches, including large language models (AUC 0.711) and clinical decision support systems (AUC 0.96) (20–22).

Substantial heterogeneity was observed (*I*^2^ = 81.48% for AUC). Meta-regression identified key sources of variation. Geographic origin was the strongest modifier (LRT *χ*^2^ = 13.51, *p* < 0.001), with Chinese studies showing higher sensitivity (0.87, 95% CI 0.83–0.91) but lower specificity (0.87, 95% CI 0.76–0.98). This pattern likely reflects the advantages of large-scale, high-volume pediatric cardiac centers in China, supported by substantial investment and extensive clinical datasets, allowing richer feature engineering and optimized model tuning tailored to local practice patterns (23).

Study size was also a significant source of heterogeneity (LRT *χ*^2^ = 6.12, *p* = 0.05). Smaller studies showed lower sensitivity (0.73, 95% CI 0.65–0.80), consistent with the well-described small-study effect in systematic reviews of diagnostic accuracy, wherein smaller cohorts tend to report inflated or more variable estimates due to selection and reporting pressures (24). In the context of machine learning, limited sample sizes additionally increase susceptibility to overfitting, where models capture noise rather than generalizable signal, thereby reducing external validity (25).

The definition of the outcome (any-stage vs. severe AKI) was a strong clinical modifier. Studies predicting any-stage AKI had higher sensitivity (0.81, 95% CI 0.71–0.91) but lower specificity (0.81, 95% CI 0.79–0.83) compared with studies predicting severe AKI (sensitivity 0.78, 95% CI 0.66–0.91; specificity 0.96, 95% CI 0.95–0.96). This difference highlights the trade-off between detecting milder events early vs. identifying high-stakes severe AKI with greater precision. Any-stage AKI prediction enables early preventive strategies such as fluid optimization, avoidance of nephrotoxic agents, and intensified monitoring to halt progression. In contrast, severe AKI prediction supports timely escalation to renal replacement therapy and aggressive hemodynamic support, potentially reducing mortality and prolonged ICU stay (3, 9).

In the exploratory subgroup analysis, no single algorithm demonstrated clear superiority across all performance metrics. RF yielded the highest pooled specificity (0.97, 95% CI 0.91–0.99) among all evaluated algorithms, suggesting a strong capacity to correctly identify patients who will not develop AKI, which could help reduce unnecessary nephroprotective interventions (12, 26). However, this estimate is based on only two studies and should be interpreted with particular caution. LR showed a broadly balanced profile with pooled sensitivity of 0.81 (95% CI 0.71–0.88) and specificity of 0.87 (95% CI 0.66–0.96). LR's relative stability in low-sample settings and its inherent resistance to overfitting nonetheless make it a methodologically defensible choice for pediatric cohorts where training data are limited (27). SVM yielded the highest pooled sensitivity (0.82, 95% CI 0.62–0.93). This makes it especially useful in high-stakes screening settings, where failing to detect a true AKI case has more serious consequences than a false positive result (28).

XGBoost showed a modest profile (sensitivity 0.74, 95% CI 0.60–0.85; specificity 0.79, 95% CI 0.76–0.82), while CatBoost displayed extreme uncertainty in sensitivity (0.75, 95% CI 0.37–0.93), making any conclusions premature. LightGBM presents an interesting paradox: Although it was the most frequently chosen best-performing model across the studies, it showed the lowest pooled sensitivity in the subgroup analysis (0.72, 95% CI 0.56–0.84). This discrepancy probably stems from its high sensitivity to hyperparameter tuning and class imbalance handling. These factors become major sources of heterogeneity when studies do not report their operating thresholds (15).

Common preoperative predictors were basal creatinine, age, eGFR, weight, and gender. These results align with Van den Eynde et al., who identified younger age, lower body weight, lower preoperative creatinine, higher eGFR, higher RACHS-1 (Risk Adjustment for Congenital Heart Surgery-1) score, longer surgery, bypass and cross-clamp times, and increased red blood cell transfusion volume as key risk factors for CSA-AKI in pediatric (29). Such predictors reflect renal immaturity and limited physiologic reserve in children, increasing vulnerability to ischemic and inflammatory injury (30).

Intraoperative variables significantly associated with CSA-AKI include CPB duration, mechanical ventilation time, and operation time. Recent studies further identified prolonged operation time increases exposure to ischemia–reperfusion, systemic inflammation, and hemodynamic instability, collectively amplifying the risk of CSA-AKI (31, 32). Early postoperative variables linked to CSA-AKI include creatinine and lactate levels. These markers reflect the immediate renal and metabolic response following surgery, which can indicate early AKI development (33).

### 4.1 Clinical practice implications

Despite promising pooled accuracy, these ML models are not yet ready for routine bedside use. The primary barrier is the paucity of external validation and prospective implementation studies. When properly implemented, ML-based risk stratification could shift care from reactive to proactive (34). Real-time EHR-integrated models could flag high-risk patients in the pediatric cardiac intensive care unit (PCICU) within hours of surgery, enabling clinicians to (1) intensify renal monitoring (frequent creatinine, urine output, biomarkers), (2) avoid or dose-adjust nephrotoxic agents (e.g., NSAIDs, certain antibiotics), (3) optimize fluid and hemodynamic management, and (4) initiate renal replacement therapy earlier in severe cases (11). One CE-certified ML model using routine EHR data in adult cardiac surgery with CPB has already demonstrated real-world utility (AUROC 0.79 for AKI, 0.83 for 30-day kidney disease) and seamless hospital-system integration without additional tuning (35). Similar deployment in pediatric settings is feasible once multicenter external validation is completed.

Although this meta-analysis demonstrates promising diagnostic performance, the substantial heterogeneity must be interpreted in the context of clinical diversity in pediatric cardiac surgery, including differences in age, congenital lesion type, surgical complexity, and baseline renal function. Recent work on AKI subphenotypes highlights the limitations of aggregate analyses and emphasizes the need for future studies to evaluate how model performance varies across clinically relevant subgroups. Moreover, current models are purely associational and cannot establish causality; therefore, target trial emulation frameworks are recommended to estimate the real-world impact of ML-triggered interventions on patient outcomes (36).

### 4.2 Limitations

This meta-analysis has several limitations. First, only seven studies were included, with four originating from China, limiting statistical power and generalizability. Second, substantial heterogeneity persisted despite meta-regression, with study region, external validation, AKI definition, and sample size as key modifiers. Third, only two studies performed true external validation; performance dropped markedly in these cohorts, indicating overfitting in derivation sets. Fourth, extreme class imbalance in Tong et al. (147 AKI vs. 8,926 non-AKI) may have inflated specificity, although sensitivity analysis yielded consistent results. Fifth, Algorithm subgroup analyses were limited by the small number of studies. Although random-effects models were applied, between-study variance estimates are unstable with few studies. Thus, findings are hypothesis-generating and comparisons across algorithms should be interpreted cautiously. Finally, the models are associational and cannot directly guide interventions.

## 5 Conclusion

Machine learning models demonstrate promising discriminatory performance for predicting CSA-AKI in pediatric patients. However, performance was significantly lower in externally validated studies, substantial heterogeneity remained despite meta-regression, and the evidence base is geographically imbalanced. Multicenter studies with rigorous external validation, prospective design, and evaluation across clinical subphenotypes are urgently needed before these models can be safely implemented in routine perioperative care.

## Data Availability

The data analyzed in this study is subject to the following licenses/restrictions: the datasets consist of aggregated and extracted data from publicly available published studies. No original raw datasets were generated or included. Access to any underlying raw data from the included studies depends on the data sharing policies of the respective original publications, which may vary and could necessitate direct requests to the corresponding authors. Requests to access these datasets should be directed to noelmisihombing@gmail.com.
