# Supplementary material for: Machine learning models for predicting postoperative acute kidney injury in pediatric cardiac surgery: a systematic review and meta-analysis
Source: Front Cardiovasc Med. 2026 Jun 18;13:1808152. doi: 10.3389/fcvm.2026.1808152 (PMC13322801; doi:10.3389/fcvm.2026.1808152)
Supplement: Supplementary file 1 [file Supplementaryfile1.docx]

Date 10 Jan 2026

Table S1. Full search string

| Database | Search Syntax | Studies |
| --- | --- | --- |
| Pubmed | ("artificial intelligence" OR "machine learning" OR "deep learning") AND ("pediatric" OR "child" OR "children") AND ("cardiac surgery" OR "heart surgery" OR "cardiopulmonary bypass") AND ("acute kidney injury" OR "renal failure" OR "AKI"). | 18 |
| Springer | ("machine learning" OR "deep learning" OR "artificial intelligence" OR "random forest" OR XGBoost OR LightGBM OR "gradient boosting") AND (pediatric OR child OR children OR infant) AND ("cardiac surgery" OR "heart surgery" OR "cardiopulmonary bypass" OR "congenital heart surgery") AND ("acute kidney injury" OR AKI OR "CSA-AKI" OR "renal failure") | 157 |
| ScienceDirect | ("machine learning" OR "deep learning") AND (pediatric OR children OR infant) AND ("cardiac surgery" OR "cardiopulmonary bypass") AND ("kidney injury" OR "renal failure") | 103 |
| DOAJ | machine learning AND pediatric OR children AND cardiac surgery | 19 |


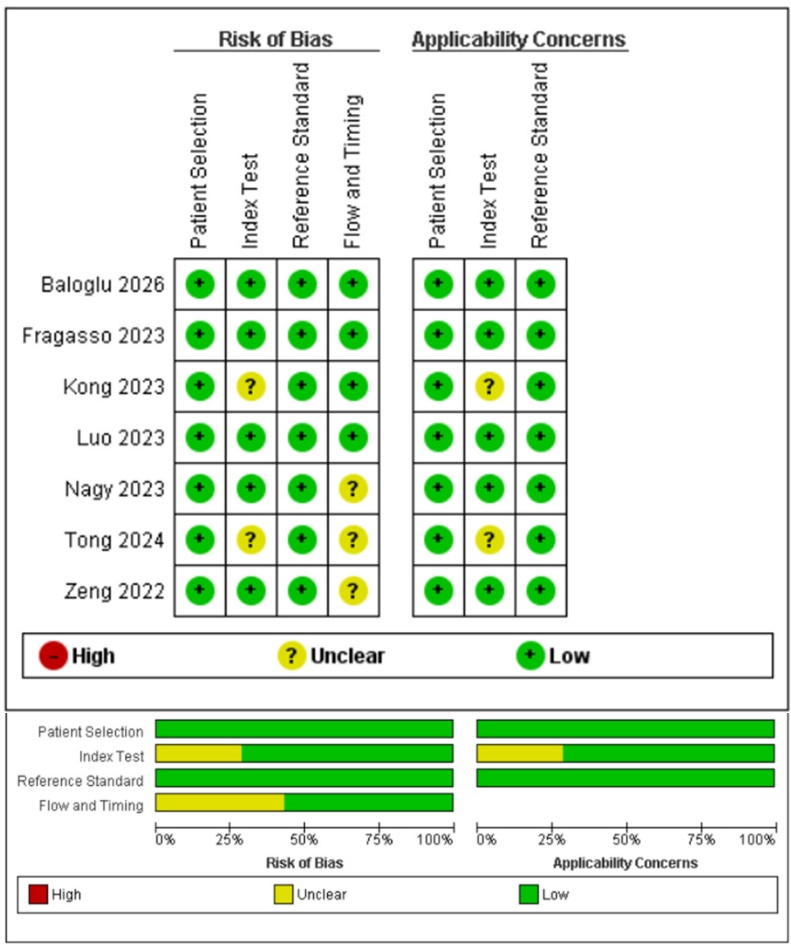


Supplementary Figure 1. QUADAS 2 to assess the risk of bias among studies.

The risk‐of‐bias assessment using the QUADAS‐2 tool is summarized in Figure 3. Overall, most domains demonstrated a low risk of bias: all studies clearly applied the index test and reference standard under appropriate conditions, and concerns regarding applicability were minimal across all domains. Notably, ‘Patient Selection’ and ‘Flow and Timing’ were rated as unclear in some studies due to limited reporting of enrollment methods.


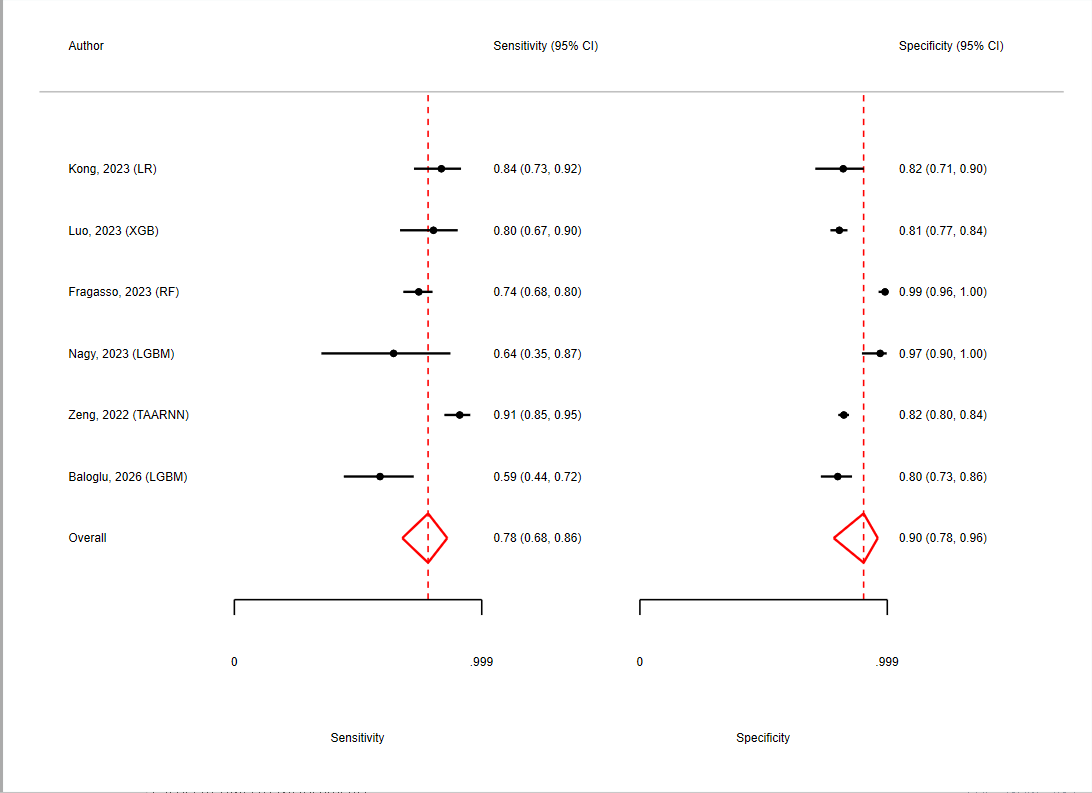


Supplementary Figure 2. Pooled sensitivity and specificity (exclude Tong et al.)

Table S2. Comparison of primary analysis (best model) vs sensitivity analysis (median model)

| Metric | Best Model | Median Model |
| --- | --- | --- |
| Sensitivity | 0.80 (95% CI 0.71–0.87) | 0.74 (95% CI 0.67–0.81) |
| Specificity | 0.91 (95% CI 0.82–0.96) | 0.91 (95% CI 0.80–0.96) |
| DOR | 42.6 (95% CI 16.95–107.10) | 28.52 (95% CI 10.05–80.96) |
| LR+ | 9.35 (95% CI 4.32–20.24) | 8.01 (95% CI 3.47–18.48) |
| LR– | 0.21 (95% CI 0.15–0.32) | 0.28 (95% CI 0.21–0.38) |
| Beta | 0.64, p = 0.166 | 1.04, p = 0.033 |
| SROC AUC | 0.91 (95% CI 0.88–0.93) | 0.85 (95% CI 0.82–0.88) |

Table S3. Comparison of primary analysis (best model) vs sensitivity analysis (median model)

| Metric | Internal validation (n=5) | External validation (n=2)* |
| --- | --- | --- |
| Sensitivity | 0.84 (95% CI 0.77–0.89) | 0.70 (95% CI 0.46–0.87) |
| Specificity | 0.95 (95% CI 0.86–0.98) | 0.80 (95% CI 0.77–0.83) |
| DOR | 97.71 (95% CI 42.04–227.10) | 9.72 (95% CI 3.33–28.42) |
| LR+ | 16.46 (95% CI 6.03–44.88) | 3.60 (95% CI 2.58–5.01) |
| LR– | 0.17 (95% CI 0.12–0.24) | 0.37 (95% CI 0.17–0.76) |
| SROC AUC | 0.93 (95% CI 0.90–0.95) | - |
| *External validation were analyzed using a univasriate random effects model (REML). | | |
